# Rapid transient and longer-lasting innate cytokine changes associated with adaptive immunity after repeated SARS-CoV-2 BNT162b2 mRNA vaccinations

**DOI:** 10.3389/fimmu.2023.1292568

**Published:** 2023-11-27

**Authors:** Margherita Rosati, Evangelos Terpos, Philip Homan, Cristina Bergamaschi, Sevasti Karaliota, Ioannis Ntanasis-Stathopoulos, Santhi Devasundaram, Jenifer Bear, Robert Burns, Tina Bagratuni, Ioannis P. Trougakos, Meletios A. Dimopoulos, George N. Pavlakis, Barbara K. Felber

**Affiliations:** ^1^ Human Retrovirus Pathogenesis Section, Center for Cancer Research, National Cancer Institute at Frederick, Frederick, MD, United States; ^2^ Department of Clinical Therapeutics, School of Medicine, National and Kapodistrian University of Athens, Athens, Greece; ^3^ Center for Cancer Research Collaborative Bioinformatics Resource, Frederick National Laboratory for Cancer Research, Leidos Biomedical Research, Inc., Frederick, MD, United States; ^4^ Basic Science Program, Frederick National Laboratory for Cancer Research, Leidos Biomedical Research, Inc., Frederick, MD, United States; ^5^ Department of Cell Biology and Biophysics, Faculty of Biology, National and Kapodistrian University of Athens, Athens, Greece; ^6^ Center for Cancer Research, National Cancer Institute at Frederick, Frederick, MD, United States

**Keywords:** Innate immunity, IL-15, IFN-g, CXCL10/IP10, TNF-a, CXCL13, myeloid cell biomarkers, anti-spike neutralizing antibody

## Abstract

**Introduction:**

Cytokines and chemokines play an important role in shaping innate and adaptive immunity in response to infection and vaccination. Systems serology identified immunological parameters predictive of beneficial response to the BNT162b2 mRNA vaccine in COVID-19 infection-naïve volunteers, COVID-19 convalescent patients and transplant patients with hematological malignancies. Here, we examined the dynamics of the serum cytokine/chemokine responses after the 3^rd^ BNT162b2 mRNA vaccination in a cohort of COVID-19 infection-naïve volunteers.

**Methods:**

We measured serum cytokine and chemokine responses after the 3^rd^ dose of the BNT162b2 mRNA (Pfizer/BioNtech) vaccine in COVID-19 infection-naïve individuals by a chemiluminescent assay and ELISA. Anti-Spike binding antibodies were measured by ELISA. Anti-Spike neutralizing antibodies were measured by a pseudotype assay.

**Results:**

Comparison to responses found after the 1^st^ and 2^nd^ vaccinations showed persistence of the coordinated responses of several cytokine/chemokines including the previously identified rapid and transient IL-15, IFN-γ, CXCL10/IP-10, TNF-α, IL-6 signature. In contrast to the transient (24hrs) effect of the IL-15 signature, an inflammatory/anti-inflammatory cytokine signature (CCL2/MCP-1, CCL3/MIP-1α, CCL4/MIP-1β, CXCL8/IL-8, IL-1Ra) remained at higher levels up to one month after the 2^nd^ and 3^rd^ booster vaccinations, indicative of a state of longer-lasting innate immune change. We also identified a systemic transient increase of CXCL13 only after the 3^rd^ vaccination, supporting stronger germinal center activity and the higher anti-Spike antibody responses. Changes of the IL-15 signature, and the inflammatory/anti-inflammatory cytokine profile correlated with neutralizing antibody levels also after the 3^rd^ vaccination supporting their role as immune biomarkers for effective development of vaccine-induced humoral responses.

**Conclusion:**

These data revealed that repeated SARS-Cov-2 BNT162b2 mRNA vaccination induces both rapid transient as well as longer-lasting systemic serum cytokine changes associated with innate and adaptive immune responses.

**Clinical trial registration:**

Clinicaltrials.gov, identifier NCT04743388.

## Graphical Abstract

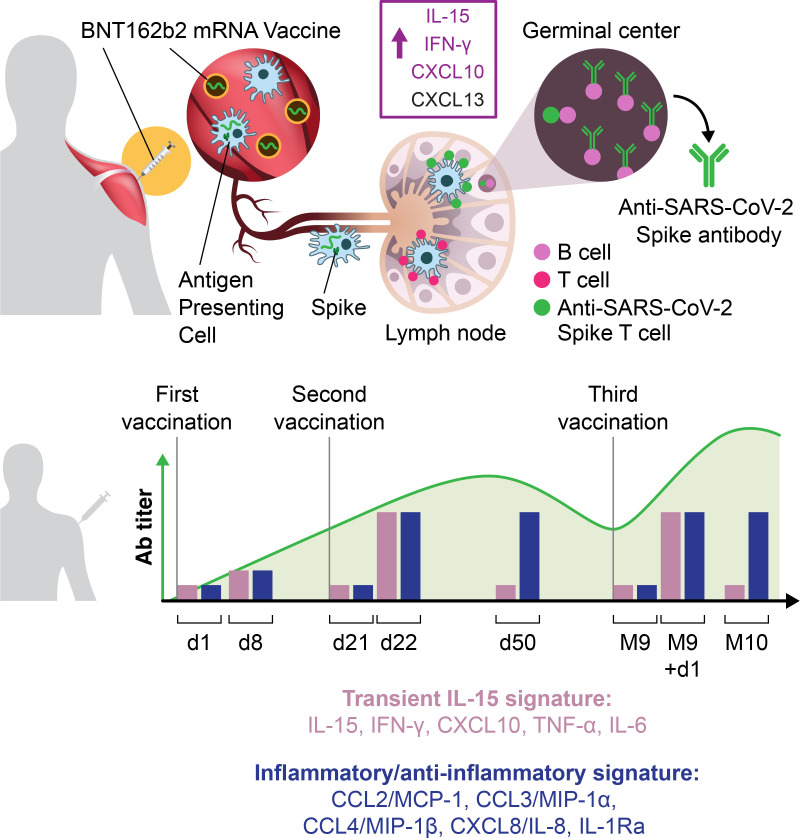

## 1 Introduction

Cytokines and chemokines play an important role in shaping innate and adaptive immunity in response to infection and vaccination. Dissecting early innate vaccine signatures may predict immunogenicity to help optimize the efficacy of mRNA and other vaccine strategies. Several studies identified immune signatures early after Yellow Fever, HIV-Ade5, HIV ALVAC, Ebola rVSV-ZEBOV, and trivalent influenza (TIV) vaccination (1–7). Understanding the effect of COVID-19 BNT162b2 mRNA (Pfizer/BioNtech) (8) vaccine on innate and trained immunity responses [reviewed in (9)] is of great interest and contributes to the modulation of adaptive immunity. We and others reported on early effects after BNT162b2 mRNA vaccination in humans using systems serology (10, 11), multi-omic approaches and flow-based assays (12–14). Systems serology identified immunological parameters predictive of beneficial response of the BNT162b2 mRNA vaccine in COVID-19 infection-naïve volunteers (10), COVID-19 convalescent patients (10) and transplant patients with hematological malignancies (11). We identified a systemic signature, detectable at 24hrs after the 1^st^ vaccination, comprising IL-15, IFN-γ, and CXCL10/IP-10, which was expanded to include tumor necrosis factor alpha (TNF-α) and IL-6 at 24hrs after the 2^nd^ vaccination (10). We further reported a correlation of changes of IL-15 and IFN-γ levels with binding antibody titers in BNT162b2 mRNA vaccine recipients (10). In contrast to vaccination of a cohort of healthy participants, patients with hematological malignancies who had a lower anti-Spike response, also had a diminished systemic cytokine response (IFN-γ, IL-15 and CXCL10/IP-10), and this correlated with the lower anti-Spike antibody levels (11). On the other hand, in patients who failed to develop antibodies, the innate systemic response showed a lack of the IL-15/IFN-γ signature with responses dominated by CCL8/IL-8 and CCL3/MIP-1α (11). Thus, we concluded that successful development of an anti-Spike immune response was associated with a robust transient activation of the IL-15 signature (10, 11). Similar to our findings after BTN162b2 mRNA vaccination, Andersen-Nissen et al. (4) reported that a different vaccine using a non-replicating vaccinia vector expressing HIV antigens (ALVAC-HIV) induced a signature of serum cytokines featuring IFN-γ, IL-15, and CXCL10/IP-10 in humans. Multi-omic approaches further reported an involvement of myeloid cells in response to the BNT162b2 mRNA vaccination in humans and mice (12–15).

In this report, we examined the dynamics of the serum cytokine/chemokine responses after the 3^rd^ BNT162b2 mRNA vaccination in a cohort of COVID-19 infection-naïve volunteers. In addition to the previously identified cytokine/chemokine responses (11), we detected transient induction of CXCL13, a biomarker for germinal center activation and a key regulator of B cells (16), found only after the 3^rd^ vaccination. In addition a set of pro-inflammatory and anti-inflammatory cytokines was detectable for up to one month post the 2^nd^ and the 3^rd^ vaccination. Together, these data revealed that repeated BNT162b2 mRNA vaccination induced both rapid/transient as well as longer-lasting cytokine/chemokine changes. Changes of some biomarkers, including IL-15, also correlated with vaccine-induced neutralizing antibody responses supporting an association of innate and adaptive immune responses.

## 2 Materials and methods

### 2.1 Study design

This is a prospective study (NCT04743388) that evaluates the kinetics of antibodies against SARS-CoV-2 as well as the kinetics of serum cytokines and chemokines associated with the immune response in volunteers receiving the BNT162b2 vaccine against SARS-Cov-2 (Comirnaty™), initiated on January 4, 2021, in Greece (10). Volunteer donors (**Table 1**) were tested in the period January to December 2021 in the vaccine center of Alexandra General Hospital in Athens, Greece. Major inclusion criteria for participation in this study included: (i) age above 18 years; (ii) ability to sign the informed consent form, and (iii) eligibility for vaccination, according to the national (Greek) program for COVID-19 vaccination (i.e., individuals who had no serious allergy problem and especially they have not been hospitalized due to a serious allergic reaction (anaphylaxis). Major exclusion criteria included the presence of: (i) autoimmune disorder under immunosuppressive therapy; (ii) active malignant disease and (iii) end-stage renal disease, as previously described (10). Individuals with documented (PCR, ELISA) prior COVID-19 infection were excluded from this report. The study recruited volunteers for vaccination 1 (n=63), vaccination 2 (n=73) and vaccination 3 (n=44). The vaccinations were administered at day 1, day 22, and month 9. The present study included frequent serum collections but did not include collection of PBMC. All study procedures were carried out in accordance with the declaration of Helsinki (18th World Medical Association Assembly), its subsequent amendments, the Greek regulations and guidelines, as well as the good clinical practice guidelines (GCP) as defined by the International Conference of Harmonization. The study was also approved by the local ethic committee of Alexandra General (no 15/23 December 2020).

**Table 1:** Demographics of vaccine recipients for Cytokine/chemokine analysis.

	Vaccination 1	Vaccination 2^1^	Vaccination 3^2^
n	63	73	44
age			
median (IQR)	50 (24)	49 (23)	53 (15)
range	28-68	28-68	28-68
gender			
men	25 (40%)	29 (40%)	15 (34%)
women	37 (60%)	44 (60%)	29 (66%)

^1^ Vaccination 1 and 2 share 62 individuals.

^2^ Vaccination 3 shares 10 individuals with cohorts of vaccinations 1 and 2.

### 2.2 SARS-CoV-2 antibodies

**Figure 1:**
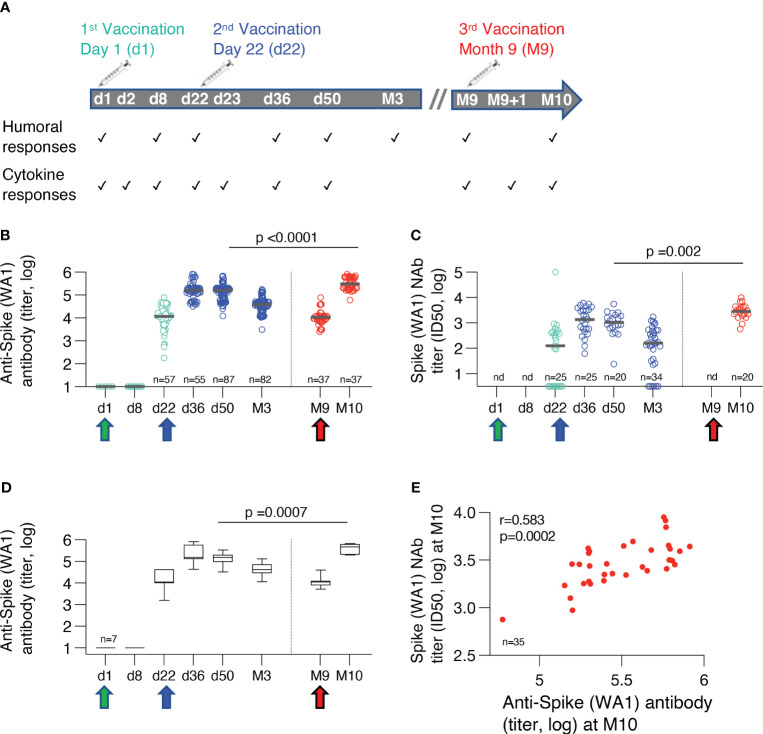
BNT162b2 mRNA cohorts and humoral immune responses. **(A)** Time points of measurements of adaptive and innate immune responses over the course of three BNT162b2 mRNA vaccinations. **(B)** Binding antibodies against ancestral trimeric Spike WA1 were measured by ELISA. Antibody titers from some individual time points have been reported previously (10, 17, 18). The Ab titers were compared at one month after the 2^nd^ (n=87) and the 3^rd^ vaccination (n=36). The p-value is from unpaired, non-parametric t test, Mann-Whitney. Median values are shown with black bars. **(C)** Neutralizing antibodies (NAb) were assessed using HIV-1_NL_ΔEnv-NanoLuc derived pseudotype virus assay carrying Wuhan-Hu-1 Spike mutant D614G. The NAb titers were compared at one month after the 2^nd^ (n=25) and the 3^rd^ vaccination (n=20). The p-value is from unpaired, non-parametric t test, Mann-Whitney. nd, not done. Median values are shown with black bars. **(D)** Binding antibodies against ancestral trimeric Spike WA1 were measured by ELISA in a subset of vaccine recipients for whom sequential samples were available for all the time points. Median values are shown with black bars. **(E)** Correlation of neutralizing (WA1 D614G) and binding Ab titers to WA1 measured at one month after the booster vaccination (Spearman r=0.583; p=0.002).

Serum was collected within 4 hours from blood collection as detailed in **Figure 1A** and stored at -80°C until the day of measurement. Sera collected after 1^st^ and 2^nd^ vaccination were part of a previous report (10). An in-house ELISA assay measuring IgG (19, 20) was used to detect Ab endpoint titers against the complete trimeric ancestral WA1/2020 Spike protein (21). The Spike pseudotyped pHIV-1_NL_ΔEnv-Nanoluc assay (22, 23) was described (17, 19) measuring eight 4-fold serial dilutions starting at 1:50. Neutralization assays were performed using 8 dilutions of heat-inactivated sera (1:40 to 1: 655,360) in triplicates in HEK293T/ACE2wt cells and luciferase levels in the cell lysates were measured. Neutralization was measured against the WA1 and Omicron BA.1 Spike as well as against the BA.5, BQ.1.1, XBB, and XBB.1.5 Spike proteins and these data were previously reported (17, 18). Ab titers and 50% Inhibitory Dose (ID50) of NAb were calculated using GraphPad Prism version 9.4.1 for MacOS X (GraphPad Software, Inc, La Jolla, CA).

### 2.3 Cytokine/chemokine analysis

Cytokine/chemokine levels (pg/ml) of 53 biomarkers were measured in sera using the chemiluminescent assay V-PLEX Human Biomarker Assay kit (Meso Scale Diagnostics LLC, Maryland, US), as listed in **Table 2**. Biomarkers which did not show changes at the time points analyzed or were below the threshold of the assay are listed. CXCL13 was measured by commercial ELISA (Invitrogen cat# EHCXCL13) using 1:2 diluted serum samples. Biomarker levels falling below the detection limit/standard range were removed if absent in more than 50% of the samples or adjusted to 50% of the lowest standard point or detection limits.

**Table 2:** Panel of cytokines and chemokines tested.

Analytes with changes (n=26)	Analytes with minimal or no change (n=11)	Analytes below detection threshold (N=17)
bFGF^1^	CCL13/MCP-4	GM-CSF
CCL2/MCP-1	CCL17/TARC	IL-12p70
CCL3/MIP-1α	CCL20/MIP-3α^1^	IL-13
CCL4/MIP-1β	CCL26/Eotaxin-3	IL-17A
CCL22/MDC	ICAM-1	IL-17A/F
CRP	IL-17B	IL-17D
CXCL8/IL-8	IL-17C	IL-1α
CXCL10/IP-10	TSLP	IL-1β
CXCL13^1,2^	VCAM-1	IL-2
Eotaxin	VEGF-C^1^	IL-21^1^
Flt-1^1^	VEGF-D^1^	IL-22^1^
IFN-γ		IL-23^1^
IL-1Ra		IL-31^1^
IL-3		IL-4
IL-6		IL-5
IL-7		IL-9
IL-10^1^		TNF-β
IL-12/IL-23p40		
IL-15		
IL-16		
IL-27^1^		
PlGF^1^		
SAA		
Tie-2^1^		
TNF-α		
VEGF-A		

^1^analytes were not reported previously for 1^st^ and 2^nd^ vaccination.

^2^all analytes were measured by the MSD platform except CXCL13 which was measured by ELISA.

### 2.4 Bioinformatics

Biomarker analysis was performed with a workflow written in R and through a user interface developed on the Foundry Platform (Palantir Technologies). Heatmaps of 26 analytes represent the log_2_ fold change in concentration between time points. Analytes with a False Discovery Rate (FDR) <0.05 were considered significantly changed. Significant changes in analyte concentrations after Vaccine 2 (d22,d23,d36,d50) and Vaccine 3(M9, M9+d1, M10) a was determined using non-parametric Anova (Friedman test). The pairwise inter-relationship between analytes were identified by calculating the Spearman correlation between analytes using the log2 fold-changes in concentration after vaccine 2 (d23 vs d22) and vaccine 3 (M9+d1 vs M9) across each patient. Analyte pairs with a Spearman’s correlation coefficient < 0.005 were considered significant relationships. Univariate correlations for analytes were identified by calculating the spearman correlation between the analytes log2 fold-change in concentration after vaccine 2 (d23 vs d22) and vaccine 3 (M9+d1 vs M9) and the corresponding NAb levels for each patient at the vaccine time point. Analytes with a Spearman’s correlation coefficient < 0.05 were considered significant.

## 3 Results

### 3.1 Study cohort

COVID-19 infection-naïve individuals were enrolled to receive three Pfizer/BioNtech BNT162b2 mRNA vaccinations between January and November 2021 (NCT04743388), administered at day 1 (n=63), day 22 (n=73) and at month 9 (M9, n=44). Cohorts receiving the two priming vaccinations and the 3^rd^ booster vaccination had similar age and gender distribution (**Table 1**). Sequential serum samples were collected on the days of vaccination (d1, d22, M9, respectively) and after each vaccination which included 24hrs later (d2, d23, M9 + 1d, respectively), as well as on d36, d50, month 3 (M3) after the 2^nd^ vaccination and one month (M10) after the 3^rd^ vaccination (**Figure 1A**).

Humoral immune responses were measured against the ancestral trimeric WA1 Spike by ELISA. Neutralizing Ab was measured using the HIV-derived pseudotype virus assay against WA1 Spike. The cross-sectional humoral response analysis showed robust responses after the 2^nd^ vaccination followed by the expected contraction of about 1 log in endpoint Ab titers over the 8 months of follow-up. Of note, this analysis showed that the 3^rd^ vaccination at M9 resulted in significantly higher Spike-specific binding (**Figure 1B**) and neutralizing antibodies (NAb) (**Figure 1C**) compared to the 2^nd^ vaccination. Similar to the cross-sectional evaluation, we made a sequential analysis of a subset of vaccinees (n=7) for whom samples were available for all the time points between the 2^nd^ and 3^rd^ vaccinations (**Figure 1D**). This analysis corroborated the cross-sectional analysis (**Figure 1B**) showing significantly higher humoral responses at M10 compared to d50, comparing the responses at one month after each vaccination. Together, the 3^rd^ vaccination provided a significant enhancement of the humoral immunity. The previously identified correlation of binding and neutralizing Ab was also maintained after the 3^rd^ vaccination (**Figure 1E**). Overall, the 3^rd^ vaccination provided a beneficial increase in humoral immunity.

### 3.2 Transient induction of a serum cytokine/chemokine profile after repeated BNT162b2 mRNA vaccination

Next, we performed a sequential analysis of serum cytokine/chemokine levels after each BNT162b2 mRNA administration both at the day of vaccination and 24hrs later (see **Figure 1A**). Sera were analyzed using the Meso Scale Discovery platform (V-plex, 54-Plex kit) and an ELISA assay (Invitrogen) to measure CXCL13. We previously reported effects in 58 participants after the 1^st^ and 2^nd^ vaccination using a smaller panel of analytes (10). The current analysis included a larger dataset with 7 additional biomarkers, including CXCL13, IL-10 and IL-27 (**Table 2**) after the 2^nd^ and 3^rd^ vaccination, including 62 participants after the 1^st^ vaccination, 72 participants after the 2^nd^ vaccination (of which 62 were also analyzed after the 1^st^ vaccination) and 44 participants after the 3^rd^ vaccination (of which 10 were also analyzed after vaccinations 1 and 2) (**Table 1**).

**Figure 2:**
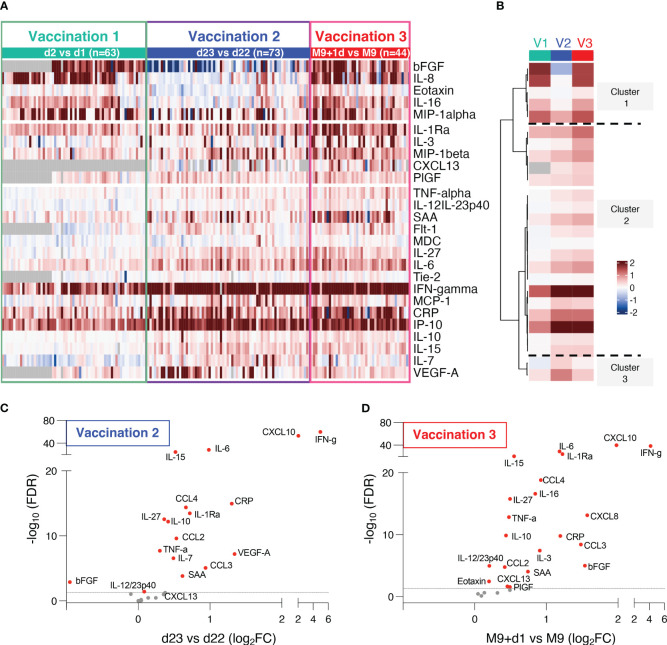
Serum cytokine and chemokine levels after BNT162b2 mRNA vaccination. **(A, B)** The heatmap **(A)** shows comparison of log2 fold changes of 26 cytokines and chemokines measured after the 1^st^ and 2^nd^ and 3^rd^ vaccination. Changes compared samples collected 24hrs after vaccination to the respective day of vaccination (d2 vs d1, d23 vs d22, M9+d1 vs M9). Grey cells denote missing values for five analytes not measured for 22 participants at d1 and d2. The analysis after the 1^st^ and 2^nd^ vaccinations includes additional data from 7 analytes (CXCL13, bFGF, Flt-1, IL-10, IL-27, PIGF, Tie-2) which were not previously reported (10). **(B)** Mean log_2_ fold changes for each analyte and each group from data shown in **(A, C, D)** Volcano plots depict differentially expressed analytes after the 2^nd^ vaccination (d23 vs d22) and **(D)** the 3^rd^ vaccination (M9d+1 vs M9) using adjusted p values. Red dots indicate significant changes (FDR<0.05) capturing 17 analytes after the 2^nd^ **(C)** and 21 analytes after the 3^rd^ vaccination **(D)**. Horizontal broken line represents FDR-adjusted p-values=0.05.

The fold changes in cytokine/chemokine levels comparing the values at the day of vaccine administration and 24hrs later were compared after the 1^st^ (d2 vs d1), 2^nd^ (d23 vs d22) and 3^rd^ (M9 + 1d vs M9) vaccinations (**Figures 2A, B**). Of the 54 analytes, 26 had significant changes over the 24-hr-follow-up, 11 analytes had no or marginal changes and 17 were below the threshold of detection (**Table 2**). Biomarkers were grouped by hierarchical clustering based on average fold change at each vaccination (**Figure 2B**). Biomarkers in cluster 1 showed no or low responses after the 2^nd^ vaccination but strong responses after the 1^st^ and 3^rd^ vaccinations; cluster 2 showed strong responses both after the 2^nd^ and 3^rd^ vaccinations, whereas cluster 3 showed a stronger response after the 2^nd^ vaccination.

**Figure 3:**
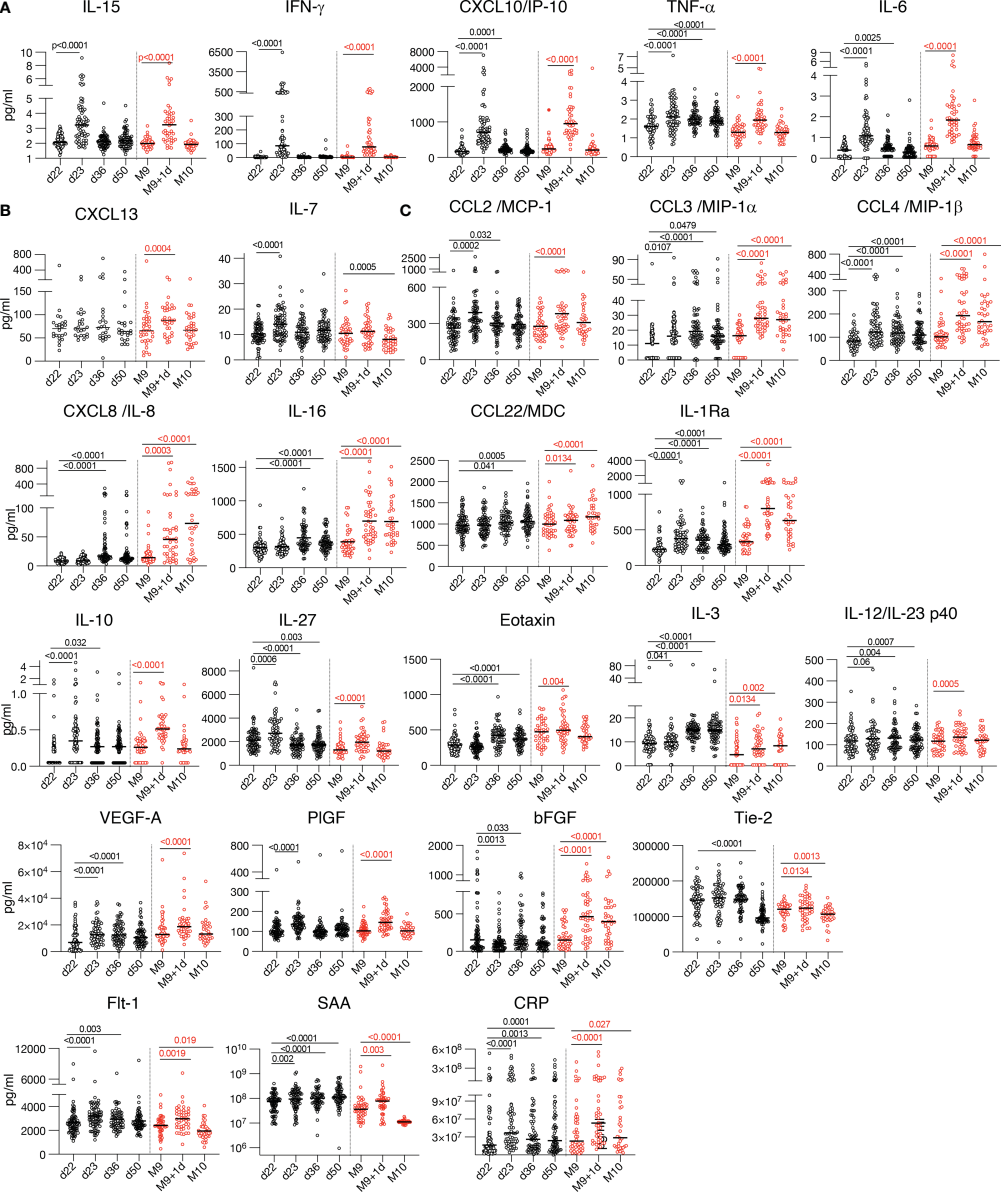
Cytokine and chemokine levels over time. Cytokine and chemokine measurements are shown over time using the Meso Scale Discovery assay and the ELISA for CXCL13 at the day of vaccination (d22, M9) and at one day after the 2^nd^ vaccination (d23) and the 3^rd^ vaccination (M9 + 1d). Additional measurements at 2 and 4 weeks (d36 and d50) after the 2^nd^ vaccination and 4 weeks (M10) after the 3^rd^ vaccination were plotted. Values (pg/ml) from individual vaccine recipients are shown after the 2^nd^ vaccination (black symbols and after the 3^rd^ vaccination (red symbols) and median values are shown (black lines). The p values (GraphPad Prism) are from Anova (Friedman test) for the 58 participants having 4 over time paired samples available after the 2^nd^ vaccination and the 36 participants having 3 over time samples after the 3^rd^ vaccination, respectively. The plots show the absolute values of all measurements: d22, d23 (n=72); d36 (n=62); d50 (n=64); M9 and M9 + 1d (n=44) and M10 (n=36). **(A-C)** Serum levels of 17 selected analytes among 26 analytes showing changes after the 2^nd^ and 3^rd^ vaccination are plotted over time: **(A)** cytokines belonging to the previously identified IL-15 signature; **(B)** Germinal center activation biomarkers CXCL13 and IL-7; **(C)** cytokine with pro-inflammatory, anti-inflammatory or dual function.

The differentially expressed biomarkers are shown in volcano plots after the 2^nd^ vaccination (**Figure 2C**) and the 3^rd^ vaccination (**Figure 2D**). Of the 26 analytes, significant changes were found in 17 analytes after the 2^nd^ vaccination, and in 21 analytes after the 3^rd^ vaccination. Absolute levels of individual biomarkers after the 2^nd^ and 3^rd^ vaccination are shown (**Figure 3**). Consistent with the rapid response after the 2^nd^ vaccination, activation of several cytokines including IL-15, IFN-γ, CXCL10/IP-10, IL-6, TNF-α, CCL4/MIP-1β, IL-1Ra, IL-10, IL-27 were detected also after the 3^rd^ vaccination.

Additionally, we further found a significant transient increase of CXCL13 only after the 3^rd^ vaccination (**Figures 2**, **3B**). CXCL13 is a biomarker for germinal center activation and a key regulator of B cells (16) [reviewed in (24)]. This finding is in agreement with the significantly higher humoral immune response found after the 3^rd^ vaccination (**Figures 1B, C**), suggesting further stimulation of B-cell development.

We also found significant increases of several other chemokines and cytokines produced by myeloid cells [reviewed in (25, 26)], including CCL2/MCP-1, CCL3/MIP-1α, CCL4/MIP-1β, CXCL8/IL-8, CCL22/MDC, and the anti-inflammatory cytokine IL-1Ra (**Figures 2**, **3**). A strong transient activation of IL-1Ra was also found. The IL-1/IL-1Ra regulatory axis plays a role in the adaptive immune response development (27, 28) with the pro-inflammatory IL-1 acting as master cytokine in inflammation (29–31). Although no systemic levels of the IL-1 cytokines, IL-1α or IL-1β, were found one day later (**Table 2**), we cannot exclude faster and transient changes of these biomarkers.

IL-12/23p40, involved in the development and modulation of adaptive responses (32), was also significantly increased (**Figures 2**, **3**). Changes in the levels of the IL-12/23p40 chain, which is part of heterodimeric IL-12p70 and IL-23 cytokines, could reflect their respective expression. Although *ex vivo* stimulation of PBMC from vaccine recipients with Spike peptide resulted in IL-12p70 expression (8), systemic serum levels of IL-12p70 as well as of IL-23 remained below the threshold of detection in mRNA/LNP vaccinated humans (**Table 2**).

The 3^rd^ vaccination also induced a significant increase of biomarkers having a pro-inflammatory role including CXCL10/IP-10, IL-16; anti-inflammatory function (IL-1Ra, IL-10, IL-3, Eotaxin) or both (IL-27) (**Figures 2**, **3**) involved in shaping the innate and adaptive immune response. Molecules released in response to inflammation [reviewed in (33)] included acute phase proteins serum amyloid A (SAA) and C-reactive protein CRP (**Figure 3**). Significant changes were found in the angiogenic cytokines VEGF-A, Flt-1, bFGF, as well as PIGF and Tie-2, two cytokines which were also reported to have macrophage activating function (34) (**Figure 3**).

Of note, a set of analytes including CXCL8/IL-8, IL-16, Eotaxin and bFGF which showed no changes after the 2^nd^ vaccination, showed significant increases within 24hrs both after the 1^st^ and the 3^rd^ vaccinations (**Figures 2A, B**, **3**). This discrepancy may be attributed, at least in part, to the short time between 1^st^ and 2^nd^ vaccination which could influence response kinetics of these biomarkers.

### 3.3 Serum cytokine/chemokine profiles showing longer-lasting innate responses after the 2^nd^ and the 3^rd^ BNT162b2 mRNA vaccinations

**Figure 4:**
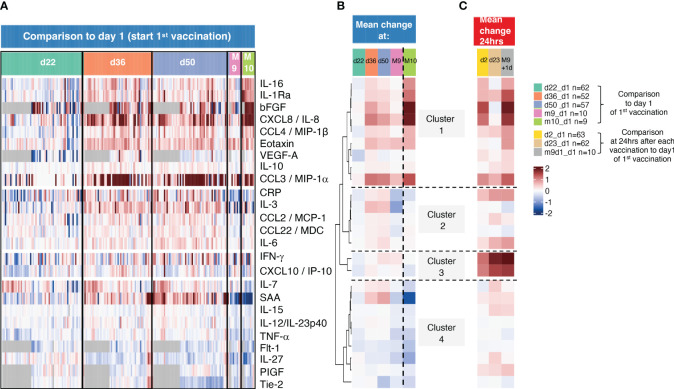
Sequential analysis of biomarkers with longer-lasting changes. **(A)** The heatmap shows log_2_ fold changes comparing all sequential measurements to the day 1 measurements before the 1^st^ vaccination. d22 samples were collected before 2^nd^ vaccination, d36, d50, and M9 before the 3^rd^ vaccination and M10, one month after the 3^rd^ vaccination. Grey cells denote missing values for five analytes not measured for 22 participants. **(B, C)** Mean log_2_ fold changes for each analyte and for **(B)** data obtained d22, d36, d50, M9 and M10 from **(B, C)** and data obtained 24hrs after the 1^st^, 2^nd^, and the 3^rd^ vaccination (d2, d23, m9+d1).

In addition to the cross-sectional analysis of the transient changes over 24hrs after each vaccination, a sequential analysis (**Figures 4A, B**) was performed. Biomarker changes were recorded from day 1 (day 1 of 1^st^ vaccination) to 3 weeks later (d22, day of 2^nd^ vaccination), to day 36 (2 weeks after the 2^nd^ vaccination), to day 50 (4 weeks after the 2^nd^ vaccination), to 8 months later at M9 (day of 3^rd^ vaccination);, and to M10 (4 weeks after the 3^rd^ vaccination). A subset of patients (n=10) had samples available for the complete time range starting at day 1 of the study, reported at M9 and M10.

Sequential changes are summarized (**Figure 4B**). By d22, all biomarkers reached baseline, as we previously observed (10). A set of biomarkers including primarily myeloid cell associated inflammatory and anti-inflammatory cytokines (CXCL8/IL-8, IL-16, CCL3/MIP-1α, CCL4/MIP-1β, IL-1Ra, Eotaxin, IL-10 and the angiogenic cytokines bFGF and VEGF-A) in clusters 1 and 2 continued to remain upregulated at d36 and d50. However, their levels were greatly reduced reaching baseline by M9 (8 months after the 2^nd^ vaccination), except CXCL8/IL-8, CCL3/MIP-1α, Eotaxin which declined to a lesser extent. The levels of these biomarkers also remained upregulated by M10, 4 weeks after the 3^rd^ vaccination (compare d50 and M10), reflecting their response kinetics after the 2^nd^ vaccination. The responses of biomarkers in cluster 2 also continued to be higher (d36, d50) but to a lower extent. In contrast, biomarkers in clusters 3 and 4 were no longer detectable at these time points in agreement with their rapid transient responses (**Figures 2**, **3**).

To compare longer-term changes to the transient changes, samples collected at 24hrs after each vaccination (d2:1^st^ vaccination, d23:2^nd^ vaccination, M9 + 1:3^rd^ vaccination) were also included in the sequential analysis comparing changes to day 1 (day 1 of 1^st^ vaccination) (**Figure 4C**). These data showed that the biomarkers in cluster 1 increased only after the 1^st^ (d2) and the 3^rd^ (M9 + 1d) vaccinations, whereas clusters 2-4 showed a stronger increase after the 2^nd^ and the 3^rd^ vaccinations with cluster 3 (IFN-γ, CXCL10/IP10) showing the highest responses, as expected (**Figures 2**, **3**). Comparison of samples from 24hrs after each vaccination (**Figure 4C**) and over time responses (**Figure 4B**) illustrated the more durable presence of a subset of biomarkers in clusters 1 and 2.

Together, these data demonstrated both a transient increase for some cytokines, as well as a longer lasting and more pronounced presence of a subset of biomarkers, persisting up to one month after the 2^nd^ and 3^rd^ vaccinations, These biomarkers primarily affect the regulation of the immune chemotaxis and myeloid cell function.

### 3.4 Correlation between cytokine changes induced by the BNT162b2 mRNA booster vaccination

**Figure 5:**
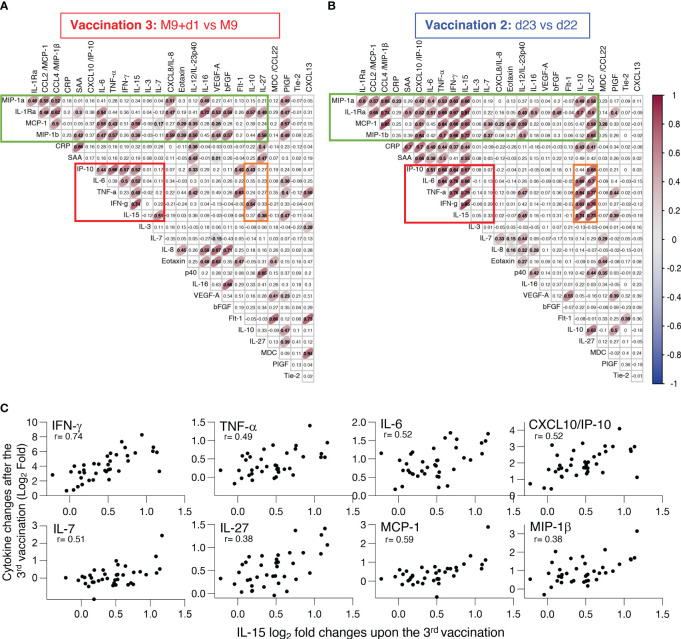
Inter-relationship of the vaccine-induced effects on different serum cytokines and chemokines. **(A, B)** Pairwise correlations were calculated between the log_2_ fold changes after **(A)** 3^rd^ vaccination (M9 + 1d vs M9) and **(B)** 2^nd^ vaccination (d23 vs d22) for the 26 biomarkers that were affected by the vaccinations using the Spearman correlation coefficient. Significant correlations (p-value < 0.005) are represented by ellipses whose color and shape correspond to the value of the Spearman correlation coefficient with red color (**Figure 2**) indicating a positive correlation. The red boxes identify the cluster of positive associations featuring IFN-γ, IL-15, TNF-α, IL-6 and IP-10/CXCL10. The green boxes denote the cluster of concerted interactions of inflammatory and anti-inflammatory cytokines. The orange boxes denote the cluster of IL-10 and IL-27 associated with the IL-15 cluster. **(C)** Scatter plots comparing the log_2_ fold change after the booster vaccination (M9+d1 vs M9) shown in panel A for analytes (IFN-γ, TNF-α, IL-6, CXCL10/IP-10, IL-7, IL-27, MCP-1, MIP-1β) significantly correlated to IL-15. Each dot represents the compared analytes log_2_ fold change for a single vaccine recipient. r and p values are shown in plots.

To assess the inter-relationship of the vaccine-induced effects on different serum cytokines and chemokines, a pairwise correlation analyses was performed using the log_2_ fold changes obtained after the 3^rd^ vaccination (M9 + 1d vs M9; **Figure 5A**). A correlation matrix of the measurements was calculated to identify significant relationships using a cut-off Spearman coefficient with a p value <0.005. The data were compared to a similar analysis performed after the 2^nd^ vaccination (10) (d23 vs d22; **Figure 5B**). Several inter-relationships among the cytokines were identified. Importantly, the 3^rd^ vaccination largely recapitulated our previous findings after the 2^nd^ vaccination (10). Comparison between the 2^nd^ and 3^rd^ vaccination showed that cytokine inter-relations after the 3^rd^ vaccination were slightly weaker, depicted with broader ellipses, and that some associations did not meet the significance requirements (**Figure 5A** vs **5B**). The fact that several biomarkers did not exhibit significant associations may reflect the lack of these interactions in general or it may be due to the time point analyzed (24hrs after the vaccination).

Despite this, a concerted and highly significant effect among IL-15, IFN-γ, TNF-α, IL-6 and IP-10/CXCL10 was observed at 24hrs after the 3^rd^ vaccination (**Figure 5A**, red box) comparable to the reported interrelation after the 2^nd^ vaccination (10) (**Figure 5B**, red box). These data confirmed that the concerted cytokine increase induced after the 1^st^ vaccination and enhanced after the 2^nd^ vaccination (10) was recapitulated after the 3^rd^ vaccination (**Figure 5**). The correlating cytokine pairs included IL-15 and IFN-γ, IL-15 and TNF-α, IL-15 and IL-6, IL-15 and IP-10/CXCL10; individual correlation plots are shown in **Figure 5C**. IL-10 and IL-27 were reported to play a role with IL-15 in NK cell biology (35–37) and were associated with all the components of the IL-15 cluster after the 2^nd^ vaccination (**Figure 5B**, orange box), these relations were weaker or lost after the 3^rd^ vaccination (**Figure 5A**, orange box). In addition, the extended panel of cytokine/chemokines used in this work revealed additional concerted interactions of the IL-15 signature cytokines with IL-10 and IL-27 after the 2^nd^ vaccination (**Figure 5B**) that were decreased after the 3^rd^ vaccination (**Figure 5A**).

The concerted inter-relationship of other myeloid cell-produced cytokines comprising of CCL3/MIP-1α, CCL4/MIP-1β, CCL2/MCP-1, TNF-α also included IL-1Ra, IL-6, and CXCL8/IL-8, IL-16. This concerted release appeared more pronounced after the 3^rd^ vaccination (**Figures 5A, B**, green boxes). This signature also associated with PIGF, a factor reported to activate monocytes, resulting in increased expression of TNF-α, IL-1Ra, CCL2/MCP-1, CXCL8/IL-8, and CCL4/MIP-1β (38). We also found that IL-15 associated with different components of this signature as well as with PIGF. This association is broader after the 3^rd^ vaccination compared to the data obtained after the 2^nd^ vaccination, although the significance was lower (depicted with wider ellipses). These data indicated a robust BNT162b2 mRNA vaccine-induced activation of myeloid cells. Interestingly, the analysis after the 3^rd^ vaccination further showed an association of TNF-α and CXCL13 (**Figure 5A**), a relation attributed to B cell recruitment (39, 40).

We also noted a link between CRP and SAA changes both after the 2^nd^ and 3^rd^ vaccination, reflecting a response to inflammation [reviewed in (33)]. On the other hand, although a series of angiogenic cytokines (VEGF-A, bFGF, Tie-1, Flt-1), reflecting processes of endothelial injury and repair, greatly increased after vaccination, no concerted interactions were found under the condition of this analysis.

In summary, the 3^rd^ vaccination resulted in a coordinated release of several biomarkers, including the IL-15 signature cytokines, which are likely responsible for the recruitment and mobilization of different immune cell subsets, supporting regulated priming and activation of immune responses.

### 3.5 Associations of components of IL-15 signature and of the inflammatory/anti-inflammatory cytokine signature with anti-Spike antibodies

**Figure 6:**
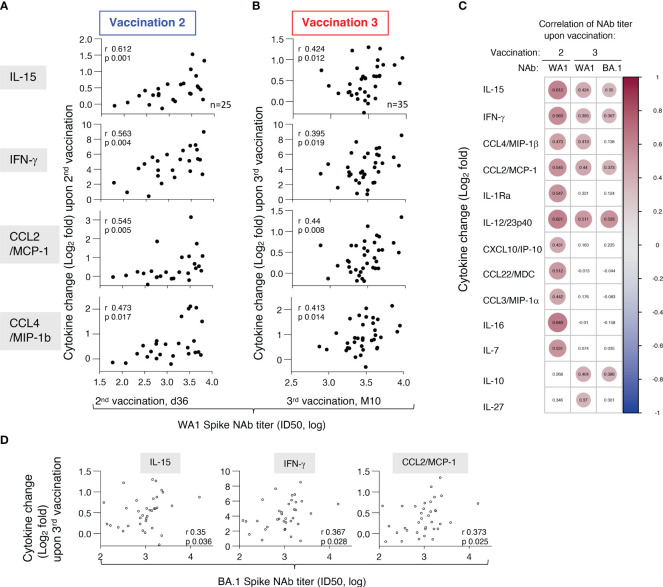
Association of biomarkers and vaccine-induced humoral immune responses. Univariate correlations of log2 fold changes of selected cytokines (IL-15, IFN-γ, CCL2/MCP-1, CCL4/MIP-1β, IL-1Ra) after the **(A)** 2^nd^ vaccination (d23 vs d22) and **(B)** 3^rd^ vaccination (M9 + 1d vs M9) and levels of anti-Spike (WA1) NAb, measured at d36 and M10, respectively. Spearman r and p values are given (calculated in R). Note the different scales of the X-axis after the 2^nd^ and 3^rd^ vaccination, with a tighter spread of NAb responses after the 3^rd^ vaccination. **(C)** Pairwise Spearman correlations for analytes with positive correlations between cytokine fold changes and NAb titers against WA1 after the 2^nd^ and 3^rd^ vaccination and NAb titers against BA.1 after the 3^rd^ vaccination. Significant correlations (p-value < 0.05) are represented by circles whose size and coloring correspond to the value of the Spearman correlation r values (calculated in R). **(D)** Univariate correlations of log2 fold changes of selected cytokines (IL-15, IFN-γ, CCL2/MCP-1) after the 3^rd^ vaccination (M9 + 1d vs M9) and levels of anti-Spike BA.1 NAb, measured at M10. Spearman r and p values are given (calculated in R).

To identify biomarkers of efficient humoral responses to vaccination, we examined the relationships between alterations in these cytokines (log_2_ fold changes after the 2^nd^ vaccination d23 vs d22 and after the 3^rd^ vaccination M9 vs M9+d1) and the levels of anti-Spike (WA1) NAb, measured after the respective vaccinations. Correlations found after the 2^nd^ vaccination (**Figure 6A**) were compared to those after the 3^rd^ vaccination (**Figure 6B**). We found significant univariate correlations of both IL-15 and IFN-γ changes after both vaccination and NAb levels. Together these results show that the IL-15/IFN-γ signature continues to serve as immune biomarker for effective development of vaccine-induced humoral responses.

Additional significant correlations were identified between NAb, both after the 2^nd^ and the 3^rd^ vaccination, and with changes with CCL4/MIP-1β, CCL2/MCP-1 (**Figures 6A, B**) and IL-1Ra as well as with IL-12/23p40 (**Figure 6C**). We further found correlations of NAb levels and the pro-inflammatory IL-10, IL-7, IL-16, and IL-27 which play a role in adaptive immune response development. Some of these Spearman correlations were only found after the 3^rd^ vaccination [IL-10 (r=0.404, p 0.016); IL-27 (r=0.37, p 0.029)], while others were only found after the 2^nd^ vaccination [IL-16 (r=0.649, p 0.0004), IL-7 (r= 0.531, p 0.006)] (**Figure 6C**). These correlations support our previous observation of correlation of some of these cytokines (CCL3/MIP-1α, CCL4/MIP-1β, CCL22/MDC, IL-1Ra) and anti-Spike humoral responses (10). Interestingly, these positive correlations included several cytokines belonging to the co-expressed cluster as reported in **Figure 5** and were re-established after the 3^rd^ vaccination. The correlation with NAb after the 3^rd^ vaccination, although weaker due to the significantly lower titers (18), further extended to some of the same cytokines and NAb titers against the more closely related Omicron BA.1 variant [IFN-γ (r=0.367, p 0.028); IL-15 (r=0.35, p 0.036), CCL2/MCP-1 (r=0.373, p 0.025), IL-10 (r= 0.386, p 0.02); IL-12/23p40 (r= 0.533, p 0.0008)] (**Figures 6C, D**). In contrast, the cytokine/chemokine responses did not correlate with NAb titers against the more divergent Spike variants BA.5, BQ.1.1, XBB, and XBB.1.5. Of note, this is due to the fact that the WA1 mRNA vaccine induced NAb showed significantly lower response rates and magnitudes against these Spike variants (17) than WA1 and BA.1. Thus, the cytokine/chemokine correlations observed were most pronounced towards the vaccine-matched WA1 NAb titers.

In conclusion, our analysis showed correlations between increases of the vaccine-induced cytokine levels with NAb levels after the 2^nd^ and 3^rd^ vaccinations. We did not find such associations using the respective baseline measurements (d1, d22 or M9) of the different biomarkers and these values did not serve as predictor of the vaccine outcome. Thus, the correlations reported here reflected the effects of vaccination on the dynamics of the cytokine/chemokine responses and their effect on the humoral response. These findings emphasize the significant contributions of innate responses to the BNT162b2 mRNA vaccination in shaping adaptive immunity and also revealed the persistence of a coordinated responses of several cytokine/chemokines after the 3^rd^ vaccination.

## 4 Discussion

In this report, we show the induction of higher binding and neutralizing Ab after the 3^rd^ vaccination, indicating a stronger adaptive immune response compared to the 2^nd^ vaccination. This observation could reflect a benefit of the longer rest period of eight months between the vaccinations. Applying systems serology, we studied the effects of the BNT162b2 mRNA COVID-19 vaccine on key plasma cytokines and chemokines measured one day after each vaccination and up to one month after the 2^nd^ and 3^rd^ vaccinations. The systemic effects of the 3^rd^ vaccination were compared to those of the 1^st^ and 2^nd^ vaccinations, showing induction of a distinct cytokine response featuring the rapid (24hrs) transient induction of IL-15 signature, including IFN-γ, TNF-a, IL-6, CXCL10/IP-10 also after the 3^rd^ vaccination. We further identified a longer lasting inflammatory/anti-inflammatory signature (>1 month). Importantly, we also found induction of biomarkers for germinal center activation including CXCL13, IL-6, IL-7, supporting the robust humoral immune response after the 3^rd^ vaccination.

We measured the changes in biomarker levels and the inter-relationship of these changes identifying clusters of biomarkers with concerted changes. This analysis revealed two major clusters including a cluster of IL-15, IFN-γ, CXCL10/IP-10, IL-6, and TNF-α and a cluster of pro-and anti-inflammatory cytokines including CCL2/MCP-1, CCL3/MIP-1α, CCL4/MIP-1β, TNF-α, IL-1Ra, IL-6, CXCL8/IL-8, IL-16. While a rapid transient change within 24hrs after vaccination was found for the IL-15 cluster, several members of the inflammatory/anti-inflammatory cytokine cluster (CXCL8/IL-8, IL-16, CCL22/MDC, Eotaxin) showed more long-lasting elevated levels, detectable up to one month after the 2^nd^ and 3^rd^ vaccination.

Correlation of several immunological parameters, including IL-15 and IFN-γ with neutralizing antibody titers indicated their positive association with humoral immune response development. In addition, the discovery of systemic CXCL13 induction indicated a stronger effect of lymph node activation after the 3^rd^ vaccination. CXCL13, also known as B-cell attracting chemokine-1 (BCA-1), plays a critical role in activating T follicular helper (Tfh) cells, which are essential contributors to B cell proliferation, differentiation, and high-affinity antibody synthesis and are required for germinal center (GC) formation and maintenance (41, 42). Other biomarkers supporting LN activity are IL-7, IL-21, IL-6, IL-4 and IL-23, cytokines involved in the development and modulation of adaptive responses and Tfh function [ (43); reviewed in (41, 42, 44)]. Systemic IL-2, IL-4, and IL-23 levels remained below the threshold of detection (**Table 2**), whereas the transient increase of IL-7 after the 3^rd^ vaccination did not reach significance, which is in contrast to its significant increase after the 2^nd^ vaccination. IL-6, readily induced after each mRNA vaccination, was reported to contribute to Tfh cell differentiation in mice (43). Together, the increases of CXCL13, IL-6 and IL-7 further support activation of LN events. In addition, others reported that additional vaccinations resulted in increase and expansion of the memory B-cells which could be in-line with increased GC activation (45).

We also observed that CXCL8/IL-8, IL-16 and bFGF which showed significant systemic increases after the 1^st^ vaccination, failed to show any changes within 24hrs after the 2^nd^ vaccination, but were readily increased within 24hrs after the 3^rd^ vaccination. One possible explanation for this could be that the 3-week rest between the 1^st^ and 2^nd^ vaccination may have been too short. A more extended interval between the two vaccinations could potentially facilitate the development of a more robust adaptive immune responses. Indeed, the 3^rd^ vaccination resulted in significantly higher humoral responses. The length of time between vaccinations has been intensely debated, supporting the notion that a prolonged rest period could allow for stronger adaptive immune response development (46–48). The short time interval between the first two BNT162b2 mRNA vaccinations of just three weeks was due to the urgency to combat the first period of COVID-19 epidemic. In future, a 0-2-6-months and a 0-6-12-months vaccine regimen should be compared to the present schedule, for SARS-CoV-2-naïve persons, so that an optimal vaccination regimen can be selected. Based on the available data it appears that repeated vaccinations within a short period, like within three weeks, may be sub-optimal.

Our analysis of early responses was performed with sera collected 24hrs post vaccination. We cannot exclude faster or delayed kinetics of cytokine responses, which could affect our interpretation of the dataset. This could apply for biomarkers that scored below the threshold of the assay as well as positive biomarkers, which could have peak responses either before or past 24hrs and thereby also affecting the correlations or lack thereof after a certain vaccination. These issues could only be addressed by more frequent sampling or by testing the effects of mRNA vaccine in an animal model, which also would allow sampling of tissues such as lymph nodes, in addition to blood.

We found that the IL-15 cluster showed a transient rapid response after the 3^rd^ mRNA vaccination. The vaccine effect was comparable to the effect of the 2^nd^ vaccination. Importantly, changes of several members of this cluster including IL-15, IFN-γ, CXCL10/IP-10 positively correlated with neutralizing antibody titers. These data showed that our observation after the 1^st^ vaccination of the concerted change of IL-15, IFN-γ, CXCL10/IP-10, which was enriched by TNF-α and IL-6 after the 2^nd^ vaccination (10) was recapitulated after the 3^rd^ vaccination. Together with the data from the HIV-ALVAC platform by Andersen-Nissen et al. (4), these findings underscore an important early role of IL-15 as part of development of an effective vaccine. Of note, although individuals with COVID-19 infection prior to the 1^st^ vaccination were excluded from this study, we had reported that such individuals showed increased transient IFN-γ, IP-10/CXCL10, TNF-α, and IL-6 responses compared to infection-naïve persons, but only after the 1^st^ vaccination (10). Thus, these data further support our findings of similar mRNA vaccine induced transient changes of the IL-15 signature cytokines upon the 2^nd^ and 3^rd^ vaccination.

IL-15 and components of this cluster have been reported to play a role in the development of innate and adaptive immune responses. IL-15, a heterodimeric cytokine (49–51), plays a role in proliferation, survival and function of many lymphocytes [reviewed in (52)] and is produced by dendritic cells and monocytes/macrophages (53, 54) as well as from endothelial cells and stroma cells in some tissues. IL-15 contributes to the development of immune responses correlating with vaccine efficacy (55–61), affects durability (62–64) and cytotoxic activity (65–67) of the immune response. IL-15 activates IL-10 production by NK cells (35, 36) and directly stimulates lymphocytes to produce IFN-γ [reviewed in (68)]. Like the other components of the IL-15 cluster, we also found correlation of IL-10 changes with NAb levels. Macrophages release IL-15, IL-6, and TNF-α as part of the inflammatory response (69). Both IL-6 and TNF-α are biomarkers of trained immunity [reviewed in (70)] and may contribute to the stronger immune response after repeated mRNA vaccination. CXCL10/IP-10 is released in response to IFN-γ, promoting chemotaxis of CXCR3^+^ cells (71, 72). An interplay between macrophage-produced CXCL10/IP-10 and B-cell produced IL-6 driving B differentiation and association with antibody production was shown (73). Serum levels of CXCL10/IP-10 were identified as part of an innate signature associated with higher vaccine-induced antibody titers (6, 7). These data underscore our reported role of IL-15 in inducing both IFN-γ directly and DC produced CXCL10/IP-10 and CXCL9/MIG through the IFN type-2 pathway in the mouse model (67). The systemic increase of IL-15, IFN-γ, CXCL10/IL-10, TNF-α and IL-6 and associated biomarkers and their concerted increase after BNT162b2 mRNA vaccination are in good agreement with other reports (12–14). Others reported associations of myeloid cells, NK cells and monocytes as well as NAb levels with changes in levels of IFN-γ, IL-6 and CXCL10/IL-10 (14) and a correlation of IFN-γ with monocyte and DC (12) and systemic increase of CXCL10 and IL-10 (12, 13). In further support of IL-15 as key early mediator of activation of innate responses, treatment of humans with recombinant hetIL-15 was reported to induce IFN-γ, IL-6, IL-27, CXCL10/IP-10, CCL3/MIP-1α, CCL4/MIP-1β (74). Indeed, our work also showed a concerted increase of IL-15 and these biomarkers after the 2^nd^ and/or 3^rd^ vaccinations. In non-human primate studies, we further identified an increase of IL-15 and IL-7, which in turn acts in lymph nodes to promote CXCL13 production (75). While PBMC were not collected as part of this vaccine study, our systems serology study point to the importance of analyzing the events in lymph nodes. Such studies however need to be performed in an animal model such as non-human primates and this was not part of this project.

We also found a concerted increase of a cluster of biomarkers (CCL2/MCP-1, CCL3/MIP-1α, CCL4/MIP-1β, CCL22/MDC, CXCL8/IL-8, IL-16) associated with myeloid cells after the 3^rd^ vaccination. We previously reported correlations of changes in CCL2/MCP-1, CCL3/MIP-1α, CCL4/MIP-1β, and CCL22/MDC after the 2^nd^ vaccination and anti-Spike antibody levels. After the 3^rd^ vaccination, several of these biomarker changes (CCL2/MCP-1, CCL4/MIP-1β) continued to correlate with NAb levels. Correlation of CCL2/MCP-1 with NK and monocytes as well as NAb levels after the 2^nd^ vaccination was recently reported (14). Thus, our data showing repeated activation after mRNA vaccination of biomarkers associated with myeloid cells [(10, 11) and this report] and the detailed transcriptomics and flow work in humans reported by others (12–14) support activation of a network of chemokines and cytokines affecting innate and adaptive cellular and humoral immune responses.

These results highlight the important role of innate responses to the BNT162b2 mRNA vaccination in shaping adaptive immunity. The persistent coordinated response of several cytokine/chemokines also after the 3^rd^ vaccination provides further insight into our understanding on their contribution to innate and adaptive immune response development. Whereas recent reports showed pre-vaccination transcriptional state of the immune system (76) or CXCL10/IP-10 levels (13) as predictive for antibody responses, our systemic cytokine analysis performed pre-vaccination (day 1) and at day 22 and month 9 was correlating with antibody response levels. In contrast, it was the dynamics of biomarker changes after each vaccination which was established as predictor of anti-Spike Ab responses. We found a correlation of a distinct set of cytokine changes (IL-15, IFN-γ, CXCL10/IP-10, CCL3/MIP-1α, CCL4/MIP-1β) after BNT162b2 mRNA vaccination of antigen-naïve individuals after 1^st^, 2^nd^, and the 3^rd^ vaccination (this report). Patients with hematological malignancies showing diminished systemic cytokine response (IFN-γ, IL-15 and CXCL10/IP-10) also had correlating but lower anti-Spike antibody levels (11).

Monitoring biomarker changes up to one month after the 2^nd^ and 3^rd^ vaccinations, we were able to detect longer-lasting changes for some cytokines. In contrast to the IL-15 signature and several other biomarkers, significant higher levels of an inflammatory/anti-inflammatory cytokine cluster were still detected showing longer-lasting increases. Interestingly, we noted previously that some of these cytokines were also still detected on day 8 post the 1^st^ vaccination but declined to baseline levels by day 22, the day of the 2^nd^ vaccination. Thus, our data showed that after the 2^nd^ vaccination and the 3^rd^ vaccination, biomarkers associated with this inflammatory/anti-inflammatory signature were produced over longer time or have longer half-lives, which resulted in their prolonged presence. It is possible that this feature is associated with or favors the development of trained immunity [reviewed in (9)].

Together, our results show coordinated responses to the BNT162b2 mRNA vaccine and highlight the important role of a network of innate responses, centering on IL-15, in shaping adaptive immunity after vaccination. This study suggests that understanding the role of these biomarkers could also help the refinement of other regimens to increase vaccine efficacy.

## Data availability statement

The original contributions presented in the study are included in the article. Further inquiries can be directed to the corresponding author.

## Ethics statement

The studies involving humans were approved by Ethics Committee of Alexandra General Hospital (Ref No. 15/23 December 2020). The studies were conducted in accordance with the local legislation and institutional requirements. The participants provided their written informed consent to participate in this study.

## Author contributions

MR: Writing – review & editing, Data curation, Formal Analysis, Investigation, Visualization. ET: Conceptualization, Supervision, Writing – review & editing, Funding acquisition, Investigation. PH: Formal Analysis, Writing – review & editing, Data curation, Visualization. CB: Writing – review & editing, Investigation. SK: Writing – review & editing, Investigation. IN-S: Writing – review & editing, Investigation. SD: Writing – review & editing, Investigation. JB: Writing – review & editing, Investigation. RB: Writing – review & editing, Investigation. TB: Writing – review & editing, Investigation. IT: Writing – review & editing, Investigation. MD: Supervision, Writing – review & editing, Conceptualization, Funding acquisition, Investigation. GP: Conceptualization, Formal Analysis, Writing – review & editing, Data curation, Funding acquisition, Supervision, Visualization, Writing – original draft. BF: Conceptualization, Data curation, Formal Analysis, Funding acquisition, Supervision, Visualization, Writing – original draft, Writing – review & editing.
